# Psychological distress across the deployment cycle: exploratory growth mixture model

**DOI:** 10.1192/bjo.2021.50

**Published:** 2021-05-04

**Authors:** Oscar A. Cabrera, Amy B. Adler

**Affiliations:** U.S. Army Medical Research Directorate-West, Walter Reed Army Institute of Research, Joint Base Lewis-McChord, USA; Walter Reed Army Institute of Research, USA

**Keywords:** Trauma, military psychiatry, depressive disorders, anxiety disorders, comorbidity

## Abstract

**Background:**

Prior research has identified behavioural health outcomes as key sequelae to combat deployment. However, relatively little is known about differential patterns of change in depression or generalised anxiety linked to deployment to a combat zone. In this paper, we add to the existing trajectory literature and examine key predictive factors of behavioural health risk.

**Aims:**

The primary aim is to leverage growth mixture modelling to ascertain trajectories of psychological distress, operationalised as a coherent construct combining depression and generalised anxiety, and to identify factors that differentiate adaptive and maladaptive patterns of change.

**Method:**

Data were collected from a brigade combat team prior to a combat deployment to Afghanistan, during deployment, at immediate re-integration and approximately 2–3 months thereafter. The main outcome was measured using the Patient Health Questionnaire Anxiety and Depression Scale (PHQ-ADS).

**Results:**

Three latent trajectories were identified: a low–stable trajectory, a declining trajectory and a rising trajectory. Most individuals aligned with the low–stable trajectory. A conditional model using covariates measured during deployment showed that the low–stable trajectory differed consistently from the remaining trajectories on self-reported loneliness and non-combat deployment stressors.

**Conclusions:**

The examination of differential patterns of adaptation, to identify individuals at higher risk, is critical for the efficient targeting of resources. Our findings further indicate that loneliness may be a useful leverage point for clinical and organisational intervention.

## Background

Prior research across a range of military samples has identified depression and generalised anxiety as key behavioural health concerns.^1–3^ This research has documented important covariates of depression and/or generalised anxiety, including functional impairment,^4^ combat exposure,^4,5^ non-combat deployment stressors (for example, separation from family)^6^ and loneliness.^7^

Few studies on depression and/or anxiety have attempted to ascertain the existence of differential longitudinal trajectories, where individuals cluster into discrete trajectories representing substantively different patterns of change and adaptation over time. We are not aware of any differential trajectory studies of generalised anxiety in military personnel, and we have identified only two such studies for depression. The first study^8^ used latent class growth analysis to examine post-deployment depression trajectories among US Army National Guard personnel who had deployed to Afghanistan. The authors reported on four trajectories: (a) a ‘resistant’ trajectory, showing a low–stable pattern across time; (b) a ‘resilient’ trajectory denoting a declining symptom pattern; (c) an ‘increasing (mild)’ trajectory showing rising symptoms over time, but remaining within a mild symptom range; and (d) a ‘chronic-dysfunction’ trajectory showing consistently higher symptomatology across time.

The second study^9^ used a prospective growth mixture modelling approach to examine trajectories of depression among Danish military personnel deployed to Afghanistan. The authors identified three trajectories of change: (a) a ‘low–stable’ trajectory with few depression symptoms; (b) a ‘low–increasing’ trajectory showing a significant rise in symptomatology over time, leading to severe depression at the last measurement occasion; and (c) a ‘medium–fluctuating’ trajectory with a pattern denoting symptomatology in the mid-range between low and severe depression. A consistent finding from these studies is that a low–stable trajectory related to depression is the most common pattern.

## Psychological distress and study objectives

Given the documented strong association between depression and generalised anxiety and that these constructs may share an underlying common factor,^10,11^ it is important to consider both of them together as they may reflect an underlying construct of emotional distress. Examining depression and anxiety together is important not only because of their comorbidity,^10–12^ but because clinical therapies for these disorders tend to be transdiagnostic^13^ and having both depression and anxiety places individuals at greater risk of symptom chronicity and may cost more in terms of resources.^14^ Moreover, individuals with one set of symptoms are at risk for developing the other, suggesting that focusing only on one construct might indicate recovery when in fact the symptom picture might simply have shifted, not abated.^15^

Indeed, it is the covariation between depression and anxiety that inspired the development of the Patient Health Questionnaire Anxiety and Depression Scale (PHQ-ADS),^13^ an instrument that combines two validated scales, the Patient Health Questionnare-9 (PHQ-9)^16^ and the Generalized Anxiety Disorder-7 (GAD-7),^17^ to assess a consolidated depression–anxiety construct. In practice, this scale has been advanced as a measure of psychological distress that may help to identify individuals at higher behavioural health risk.^18,19^ Consequently, the conceptual and methodological orientation of considering a combined depression/anxiety construct, as an index of psychological distress, presents an excellent opportunity to assess longitudinal variation in behavioural health risk.

To date, no study has examined latent trajectory heterogeneity in psychological distress, as defined here (i.e. as a combined depression–anxiety construct). The present study aims to close this gap. First, this study utilises growth mixture modelling to measure heterogeneity in the course of psychological distress, which will facilitate the identification of differential risk groups. Second, this study ascertains the modal latent trajectory pattern (i.e. the pattern with which the majority of individuals align). In this way, we will be able to better understand the most common experience of service members. Third, this study includes predictors of assignment-to-trajectory included in the depression trajectory studies cited above (i.e. combat exposure and rank), as well as important predictors not previously included (i.e. loneliness and non-combat deployment stressors). Finally, these predictors are all measured during deployment, enabling an assessment of important covariates temporally linked to the combat experience.

## Method

### Participants

Survey data were collected from soldiers serving in a brigade-sized combat unit deployed to Afghanistan in the 2013–2014 time frame, as part of a larger study of health and resilience across the deployment cycle.^20,21^ Data on the PHQ-9 and the GAD-7 were only available for four time points. Thus, the final analytic sample (*n* = 1142) consisted of those soldiers who deployed with the unit to Afghanistan, who provided complete data on the outcome measure at time 2 (during deployment), and who provided complete data on at least one of the other three sessions: (a) time 1 (pre-deployment); (b) time 3 (initial post-deployment phase, approximately 1 month after returning home); and (c) time 4 (approximately 2–3 months post-deployment). This selection was affected to maximise sample sizes at time 2, where the time code for model intercept was set because of the intended use of predictors co-occurring with the unit's deployment to Afghanistan. Demographic characteristics of the analytic sample from each time period are presented in Table 1. Demographic characteristics of the sample not selected for analysis are presented in Supplementary Table 1 available at https://doi.org/10.1192/bjo.2021.50.

**Table 1:** Demographics^a^^,^^b^

	Time 1 (*n* = 760)	Time 2 (*n* = 1142)	Time 3 (*n* = 1142)	Time 4, *n* (%) (*n* = 547)
Age, years^c^				
18–24	422 (55.5)	556 (48.7)	516 (45.2)	239 (43.7)
25–29	202 (26.6)	326 (28.5)	354 (31.0)	170 (31.1)
30–39	120 (15.8)	213 (18.7)	223 (19.5)	111 (20.3)
40 and older	16 (2.1)	44 (3.9)	47 (4.1)	26 (4.8)
Missing data	–	3 (0.3)	2 (0.2)	1 (0.1)
Gender				
Men	717 (94.3)	1070 (93.7)	1080 (94.6)	526 (96.2)
Women	37 (4.9)	55 (4.9)	58 (4.8)	20 (3.7)
Missing data	6 (0.8)	17 (1.5)	4 (0.3)	1 (0.1)
Rank^c^				
Junior enlisted	441 (58.0)	611 (53.5)	571 (50.0)	254 (46.4)
Non-commissioned officer	236 (31.1)	396 (34.7)	442 (38.7)	223 (40.7)
Officer/warrant officer	80 (10.5)	126 (11.0)	127 (11.1)	70 (12.8)
Missing data	3 (0.4)	9 (0.8)	2 (0.2)	–

a. The percent of the analytic sample taken from the total deployed sample was: 760/1125 (68%) at time 1; 1142/1186 (96%) at time 2; 1142/1936 (59%) at time 3; and 547/784 (70%) at time 4.

b. Percentages are rounded up.

c. Changes in proportions for age and rank are normative or consistent with the military promotion tempo, and expected given the over 11 months between the first and last assessments for these analyses.

Soldiers were tracked within their units, and some participant loss was expected. Specifically, attrition was predictable given that military service is characterized by substantial geographic mobility, and that some soldiers would be unable to participate in specific data collection occasions because of conflicting duty assignments. Indeed, as shown in Table 1, there was participant loss over time, although it is important to note that the loss documented in this study is consistent with similar attrition reported in other studies that have examined units across their deployment cycle.^22,23^

The study was conducted under a human-use protocol approved by the Institutional Review Board of the Walter Reed Army Institute of Research (WRAIR). The investigators have adhered to the policies for protection of human participants as prescribed in AR 70–25. Participants provided informed consent prior to enrolment, with 92% consenting to participate.

### Measures

The outcome was measured using the 16-item PHQ-ADS.^13^ As noted previously, the PHQ-ADS combines depression and anxiety items from the PHQ-9^16^ and the GAD-7,^17^ and the psychometric properties of the PHQ-ADS as a combined measure have been explored and validated with various samples. Items were rated in terms of the past month on a four-point scale (‘not at all’ to ‘nearly every day’). The possible range was from 0 to 48. For reference, cut-off scores for mild, moderate, and severe categories are 10, 20 and 30 on this scale. Coefficient alpha was 0.93, 0.93, 0.92, and 0.92 across the four time points.

Combat exposure (time 2) was measured using 22 items adapted from the WRAIR Combat Exposure Scale.^2,4^ Sample items included: ‘receiving small arms fire’; ‘handling or uncovering human remains’. On each question, the participant was asked the number of times they had experienced that event on combat deployments since 9/11 (‘never’ to ‘five or more times’). To simplify analyses, response options were dichotomised (‘yes’/‘no’) and the resulting items were summed to create the combat exposure construct. Combat exposure items were considered formative, not reflective, so coefficient alpha for this measure was not calculated.^24^

Loneliness (time 2) was measured with a nine-item scale adapted from the UCLA Loneliness Scale.^25,26^ A list of items related to loneliness were presented, and respondents were asked how often they felt that that aspect of loneliness applied to them. Sample items included: ‘how often to do you feel that you lack companionship?’; ‘how often to do you feel close to people?’; etc. The response scale ranged from 1 (‘never’) to 4 (‘always’). Positively worded items were reverse-coded, such that higher scores for all items indicated stronger feelings of loneliness. Cronbach's alpha for this scale was 0.90.

Non-combat deployment stressors were measured using 12 items from a scale developed by the WRAIR, and used in behavioural health assessments and published studies with deployed troops.^5,27^ Respondents were asked to rate how much ‘trouble or concern’ had been caused by a list of stressors. Examples of stressors that individuals could endorse included the following: ‘being separated from family’; ‘lack of privacy or personal space’. Response options ranged from 1 (‘very low’) to 5 (‘very high’) and 6 (‘does not apply’); this last value was set to missing and items were summed. Cronbach's alpha for the scale was 0.86. Supplementary Table 2 shows the array of non-combat deployment stressors, with associated endorsement rates.

Rank (time 2) was measured using one item embedded within the demographics section of the survey. Categories for this variable were ‘junior enlisted’, ‘non-commissioned officer (NCO)’, and ‘officer/warrant officer’. To facilitate group contrasting, this variable was dichotomised ‘junior enlisted’ and ‘other’.

### Analytic plan

Growth mixture analyses were executed following recommendations to conduct initial enumeration of trajectories within an unconditional model (i.e. omitting covariates), with subsequent integration of covariates in a final, conditional model.^28–33^ As noted earlier, the model intercept was set at time 2, during deployment. Missing data were expected and were the result of random variation in the military re-assignment cycle and/or unit operational requirements during data collections. Thus, data were assumed to be missing-at-random (MAR) and full information maximum likelihood was employed in all analyses. This method has been shown to yield unbiased parameter estimates under the MAR assumption.^34,35^

For unconditional model selection, three major criteria were used: (a) interpretability and parsimony; (b) Bayesian information criterion (BIC) and the bootstrap likelihood ratio test (BLRT); and (c) entropy. With regard to statistical criteria, prior simulation work^36^ has found that BIC performed better than other information criteria in identifying the correct number of classes, and the BLRT held the advantage in correct class enumeration when compared to other likelihood ratio test indices. Previously published guidelines were used to ascertain the magnitude of BIC changes.^37^ All analyses were carried out in MPlus, Version 8.2.^38^

## Results

### Unconditional model

Following definition of model selection criteria, we proceeded to estimation of the unconditional model. Here, attempts to fit a quadratic term encountered convergence errors, suggesting simpler patterns fit the data better. Therefore, we modified the model to estimate intercept and first-order slope terms only. This model configuration converged normally, and it showed that the two-class model represented an improvement over the single-class model, while the three-class model provided a better solution than the two-class model. With extraction of four trajectories, we encountered an issue involving the first selection criterion, interpretability and parsimony. Specifically, the fourth trajectory was very similar in character to one of the trajectories extracted in the three-class model: it appeared that the algorithm segmented one trajectory into a slightly lower and slightly higher variant, with the same slope. This specific type of trajectory segmentation has been identified in prior research as a warning of potential over-extraction, with the recommendation to explore solutions with fewer trajectories as a more defensible approach to model selection.^33^ Therefore, mindful of the violation of parsimony and the potential for over-extraction, we selected the three-class model as the final unconditional model. Table 2 displays fit statistics defining one- through four-trajectory models. Indices for analyses of depression and anxiety, as separate constructs, are presented in Supplementary Tables 3 and 4, respectively.

**Table 2:** Fit indices from unconditional modelling

	One class	Two classes	Three classes	Four classes (not selected)
Loglikelihood	−11 647.36	−11 429.54	−11 332.02	−11 277.04
Akaike information criterion	23 312.72	22 883.09	22 694.03	22 590.07
Bayesian information criterion	23 358.08	22 943.58	22 769.64	22 680.80
Sample size adjusted-Bayesian information criterion	23 329.50	22 905.46	22 722.00	22 623.63
Entropy	N/A	0.92	0.90	0.89
Lo-Mendell-Rubin likelihood ratio test, *P*	N/A	<0.001	<0.01	0.37
Vuong-Lo-Mendell-Rubin likelihood ratio test, *P*	N/A	<0.001	<0.01	0.39
Bootstrap likelihood ratio test, *P*	N/A	<0.001	<0.001	<0.001
Smallest class, %	N/A	12.0	7.1	4.3

NA, not applicable.

Entropy for the unconditional model was well within the acceptable range: 0.90.^33^ The largest trajectory (83.8% of respondents, ‘low–stable’) consisted of a pattern of psychological distress marked by low scores at time 2 (*b* = 3.46, s.e. = 0.19, *P* < 0.001), with negligible change in absolute scores over time (*b* = −0.33, s.e. = 0.14, *P* < 0.05). The next largest trajectory (9.1% of respondents, ‘increasing’) aligned with a pattern of moderate psychological distress at time 2 (*b* = 14.16, s.e. = 0.72, *P* < 0.001), occurring jointly with large increases in distress scores over time (*b* = 7.75, s.e. = 0.77, *P* < 0.001). The third trajectory (roughly 7.1% of respondents, ‘decreasing’) showed higher psychological distress at time 2 (*b* = 16.55, s.e. = 1.11, *P* < 0.001), followed by large decreases in distress scores over time (*b* = −7.69, s.e. = 1.65, *P* < 0.001). The averaged probabilities for assignment-to-trajectory were: 97% for the ‘low–stable’ trajectory; 84% for the ‘decreasing’ trajectory; and 90% for the ‘increasing’ trajectory.

**Fig. 1:**
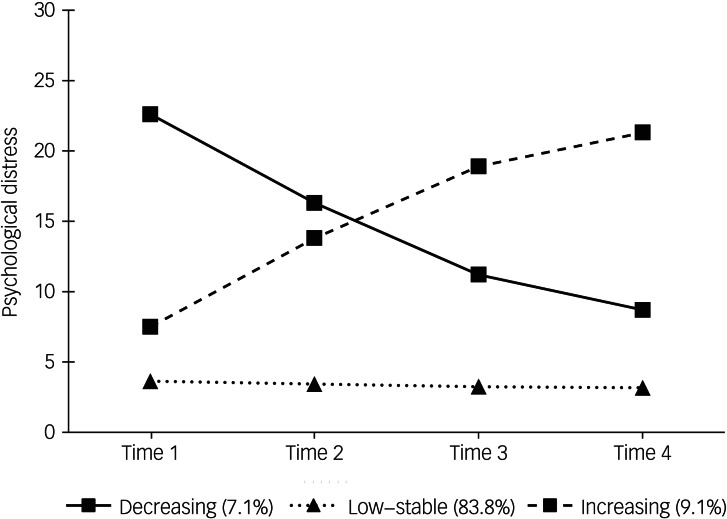
Trajectories extracted from unconditional model.

Figure 1 shows the three trajectories, as extracted from the unconditional model.

### Conditional model

For conditional model analyses, four time-invariant covariates were added in sequence. This model encountered convergence issues with the addition of a third covariate, which was resolved by constraining the slope variance (this parameter estimate was small and non-significant). As expected from this minor adjustment, the trajectories remained consistent with those defined in the previous step (see Fig. 1). There was a minor increase in entropy (0.91). The mixture changed slightly: 83.7% for low–stable; 9.8% for the ‘increasing’ trajectory; and 6.5% for the ‘decreasing’ trajectory.

Subsequently, we proceeded to examine covariate effects. These tests yielded interesting results. First, combat exposure did not differentiate trajectories. There were significant contrasts for the remaining three predictors, focused on differences between the ‘low–stable’ trajectory and the other two trajectories. Specifically, for every 1-point increase in reported non-combat deployment stressors, the odds of assignment to the ‘increasing’ and ‘decreasing’ trajectories rose significantly versus assignment to the ‘low–stable’ 'trajectory. With higher reported loneliness, the odds of assignment to the ‘increasing’ and ‘decreasing’ trajectories also increased significantly versus assignment to the ‘low–stable’ trajectory. In addition, with increase in rank, the odds of assignment to the ‘decreasing’ category versus assignment to the ‘low–stable’ category fell significantly, although the effect was weak. Finally, there were no significant differences between the ‘increasing’ and ‘decreasing’ trajectories on any of the covariates chosen for this study.

Table 3 shows the odds ratio (and 95% CIs) associated with assignment-to-trajectory when compared with the odds of assignment to the ‘low–stable’ trajectory, as a function of a 1-unit increase in the value of each predictor.

**Table 3:** Odds ratios for trajectory contrasts^a^^,^^b^

	Combat exposure, OR (95% CI)	Non-combat deployment stressors, OR (95% CI)	Loneliness, OR (95% CI)	Rank, OR (95% CI)
Low–stable	Reference	Reference	Reference	Reference
Increasing	1.03 (0.98–1.08)	**1.04 (1.01–1.07)**	**1.09 (1.05–1.14)**	0.831 (0.42–1.66)
Decreasing	1.04 (0.99–1.10)	**1.12 (1.05–1.19)**	**1.16 (1.07–1.27)**	**0.36 (0.13–0.96)**

a. Odds ratio is based on 1-unit increment in value of covariate with 95% CI shown in parentheses.

b. Bold indicates significant contrast (*P* < 0.05).

## Discussion

### General findings

The literature on differential response trajectories associated with military deployment is notable for its focus on post-traumatic stress. Less attention has been paid to depression and generalised anxiety, although two studies^8,9^ examined depression from the vantage point of latent trajectory modelling. The present study was designed to expand this area of study by examining trajectories of psychological distress, constituted as consolidated responses for depression and generalised anxiety and using the PHQ-ADS to operationalise the construct. This approach was selected in order to model a broad-based index of behavioural health risk, thereby maximising the utility of our findings for clinicians and end-users tasked with identifying military personnel at higher risk of negative sequelae across the deployment cycle. Analyses yielded three trajectories of change: a ‘low–stable’ pattern denoting low scores across time; a ‘decreasing’ trajectory showing significant reductions in psychological distress scores; and an ‘increasing’ trajectory indicative of a significant rise in psychological distress. Unlike other trajectory studies, there was no ‘chronic’ trajectory of psychological distress. This lack of a chronic subtype might be the result of greater fluctuations in depression and anxiety symptomatology over the course of time and/or may be indicative of sample-specific characteristics (i.e. reflecting health and resilience in the analytic sample).

A few conclusions can also be gleaned from these results. First, these trajectories re-capitulate some of the findings reported in the two depression trajectory studies cited above. Specifically, our study replicates two of the three trajectories from a study of Danish personnel deployed to Afghanistan,^9^ and three of the four patterns reported in a post-deployment study of National Guard personnel.^8^ We recognise that differences between our study and these other studies may be because we used a combined depression–anxiety construct rather than focusing on depression alone.

Second, the most common trajectory in this study was a low–stable pattern denoting relatively low levels of psychological distress throughout deployment, which is consistent with the depression trajectory studies cited above and with prior theoretical work.^39,40^ This finding bolsters the conclusion that low expression of this outcome is the modal response pattern across the deployment cycle.

Third, combat-related events did not differentiate among the patterns of adaptation we identified. This replicates results reported in one of the depression studies cited above,^9^ where the combat exposure construct was not a significant predictor of assignment-to-trajectory for depression. Critically, however, non-combat deployment stressors differentiated the low–stable trajectory from the remaining two trajectories. The consistent ability of non-combat deployment stressors to differentiate trajectories, above and beyond the influence of combat exposure, extends findings pioneered elsewhere,^5,6,27^ and points to the need to model a wider array of modifiable stressors in the deployed environment that may have an impact on behavioural health outcomes.

Fourth, loneliness, measured during deployment, emerged as a significant predictor of trajectory. This finding reinforces evidence of the deleterious effects of loneliness on health found in civilian^41^ and military studies.^7,42^ This finding also suggests the need for future research to identify what variables can be leveraged to reduce the risk of loneliness, including team-based interventions,^43^ the use of peer supports (for example Trauma Risk Management, or TRiM)^44^ and leadership targeting specific health-related behaviours.^20^

Taken together, the results suggest that although the majority of individuals returning from combat do not report psychological distress, there are subgroups of soldiers at risk for increasing symptomatology. Identifying this subgroup is critical for developing early interventions that can be used to flatten the rising trajectory. In contrast, individuals prior to deployment who are reporting high levels of psychological distress can be targeted for interventions to facilitate their decrease in symptoms. In both cases, such interventions can include formal treatment and/or training designed to support healthy adaptation through peer-based support and targeted leadership.^20^ Importantly, considering depression and anxiety through a lens of comorbidity is essential for addressing the fact that one set of symptoms may lead to another,^15^ that having both sets of symptoms leads to greater chronicity^12^ and this combination of symptoms is associated with greater resource utilisation.^14^ Thus, it is of benefit to consider both sets of symptoms together, rather than in isolation.

### Limitations

Three major study limitations need to be acknowledged. First, although the sample size for this analysis was fairly robust, larger sample sizes may be useful, especially given that the objective of growth mixture modelling is to dis-aggregate the overall outcome distribution into smaller components. Second, this analysis was limited to one combat deployment and may not generalise to other, more kinetic deployments or deployments that involve humanitarian response (for example, responding to a pandemic). Finally, data to create the PHQ-ADS measure were only available for four time points, and covered only up to 3 months after return from deployment. This provides a limited vantage point from which to examine the course of this phenomenon. With a longer time frame and additional time points, examination of more complex patterns of change becomes feasible, and such data may provide a better assessment of the evolution of psychological distress. Inclusion of time-varying covariates also becomes possible with additional time points.

### Implications

We hope that these findings will encourage further examination of trajectories of outcomes related to the deployment cycle in order to better understand the impact of a combat deployment on the adjustment of service members. Latent trajectory modelling with other related outcomes (for example, functional impairment, sleep problems, optimism) can be useful in understanding the way in which groups of individuals respond to this type of high-stakes occupational demand. Potential extensions of this study include examinations that extend analysis of distress across a longer time frame, preferably following units over years, to assess the lasting impact of deployment on the course of this phenomenon.

With regard to intervention development, latent trajectory studies such as this provide a better vantage point from which to identify potential targets of intervention. As an example, findings in this study about the influence of non-combat deployment stressors may represent the core of new clinical and/or organisational interventions. That is, our results suggest potential avenues for clinicians, first-line supervisors and senior military leaders to consider in establishing environmental conditions that may support the mental health, and ultimately the functioning, of deployed military personnel. For example, stressors that reflect living conditions (such as ‘lack of privacy or personal space’) could be addressed in a mature theatre of operations, and stressors that reflect uncertainty (such as ‘continuous operations’) could be addressed through establishing parameters to manage expectations. Likewise, our robust findings regarding the covariation between loneliness and psychological distress may provide a fruitful avenue for the development of clinical interventions that target this specific risk factor.

Thus, with each application of latent trajectory modelling, opportunities arise to improve our ability to have a positive and proactive impact on the health and performance of military personnel. The fact that these risk factors were assessed during deployment also suggest that interventions might be considered either prior to or during the actual deployment, which in turn may influence subsequent post-deployment adjustment. Existing behavioural health resources such as combat operational stress control teams^45^ may be able to integrate key prevention strategies and implement training with leaders or with units during critical phases. Finally, it is important to ascertain if these trajectories are directly related to the deployment cycle or if they also reflect adjustment in garrison life, as well.

## Data Availability

The data that support the findings of this study are available on request from the corresponding author. The data are not publicly available because of restrictions related to human participants protection requirements within the Institute.

## Supplementary material

For supplementary material accompanying this paper visit http://dx.doi.org/10.1192/bjo.2021.50. click here to view supplementary material
