# Supplementary material for: Psychological distress across the deployment cycle: exploratory growth mixture model
Source: BJPsych Open. 2021 May 4;7(3):e89. doi: 10.1192/bjo.2021.50 (PMC8142545; doi:10.1192/bjo.2021.50)
Supplement: Supplementary file 1 [file bjosup.zip › S2056472421000508sup001.docx]

| Table 7 (Supplementary)  Fit indices from unconditional modeling of anxiety outcome alone | | | | |
| --- | --- | --- | --- | --- |
|  | One Class | Two Classes | Three Classes^1^ | Four Classes  (Not Selected)^2^ |
| Loglikelihood | -9306.92 | -9063.21 | -8956.85 | -8874.83 |
| AIC | 18631.83 | 18150.43 | 17943.70 | 17785.67 |
| BIC | 18677.20 | 18210.91 | 18019.31 | 17876.40 |
| SSA-BIC | 18648.61 | 18172.80 | 17791.66 | 17819.22 |
| Entropy | N/A | .94 | .91 | .90 |
| LMR-LRT^3^ p-value | N/A | < .01 | < .01 | .19 |
| VLMR-LRT^4^ p-value | N/A | < .01 | < .01 | .20 |
| BLRT^5^ p-value | N/A | < .001 | < .001 | < .001 |
| Smallest Class | N/A | 10.4% | 7.9% | 3.2% |
| ^1^ Model selected met all *a priori* criteria  ^2^ Model rejected based on failure to meet “parsimony and interpretability” criterion  ^3^ Lo-Mendell-Rubin Likelihood Ratio Test  ^4^ Vuong-Lo-Mendell-Rubin Likelihood Ratio Test  ^5^ Bootstrap Likelihood Ratio Test | | | | |
