# Supplementary material for: Psychological distress across the deployment cycle: exploratory growth mixture model
Source: BJPsych Open. 2021 May 4;7(3):e89. doi: 10.1192/bjo.2021.50 (PMC8142545; doi:10.1192/bjo.2021.50)
Supplement: Supplementary file 1 [file bjosup.zip › S2056472421000508sup002.docx]

| Table 4 (Supplementary)  Demographics for non-selected respondents | | | | |
| --- | --- | --- | --- | --- |
|  | Time 1  (n = 365)^1^ | Time 2  (n = 44) | Time 3  (n = 794) | Time 4  (n = 237) |
| Age |  |  |  |  |
| 18-24 | 194 (53.3%)^2^ | 17 (38.6%) | 339 (42.9%) | 103 (43.5%) |
| 25-29 | 89 (24.5%) | 16 (36.3%) | 242 (30.6%) | 72 (30.4%) |
| 30-39 | 71 (19.5%) | 9 (20.5%) | 178 (22.5%) | 51 (21.5%) |
| 40 and Older | 10 (2.8%) | 2 (4.6%) | 32 (4.1%) | 11 (4.6%) |
| Gender |  |  |  |  |
| Male | 343 (94.8%) | 43 (97.7%) | 751 (95.4%) | 229 (97.0%) |
| Female | 19 (5.3%) | 1 (2.3%) | 36 (4.6%) | 7 (3.0%) |
| Rank |  |  |  |  |
| Jr Enlisted | 187 (54.9%) | 22 (50.0%) | 392 (49.8%) | 107 (45.2%) |
| NCO | 111 (33.5%) | 16 (36.4%) | 315 (39.9%) | 104 (43.8%) |
| Off. / Warrant Off. | 39 (11.5%) | 6 (13.6%) | 81 (10.3%) | 26 (11.0%) |
| ^1^ There were no statistically significant differences between the selected sample and the non-selected sample at any measurement occasion.  ^2^ Percentages are rounded up. Numbers may not sum to total sample size due to missing data. | | | |  |
