# Supplementary material for: Psychological distress across the deployment cycle: exploratory growth mixture model
Source: BJPsych Open. 2021 May 4;7(3):e89. doi: 10.1192/bjo.2021.50 (PMC8142545; doi:10.1192/bjo.2021.50)
Supplement: Supplementary file 1 [file bjosup.zip › S2056472421000508sup003.docx]

| Table 5 (Supplementary)  Items from non-combat deployment stressors scale | | | |
| --- | --- | --- | --- |
| Trouble or Concern Caused By: | Very Low/Low | Medium | High/Very High |
| Being separated from family | 565 (51.0%) | 332 (29.9%) | 212 (19.1%) |
| Illness or problems back home | 780 (70.9%) | 207 (18.8%) | 113 (10.3%) |
| Boring and repetitive work | 394 (35.4%) | 299 (26.8%) | 421 (37.8%) |
| Difficulty communicating back home (such as: phone calls, email, mail) | 867 (78.4%) | 172 (15.6%) | 67 (6.1%) |
| Uncertain redeployment date | 667 (60.8%) | 227 (20.7%) | 204 (18.6%) |
| Lack of privacy or personal space | 596 (53.5%) | 247 (22.2%) | 272 (24.3%) |
| Lack of time off for personal time | 486 (43.7%) | 293 (26.4%) | 332 (29.9%) |
| Not having right equipment or repair parts | 738 (67.5%) | 221 (20.2%) | 134 (12.3%) |
| Not getting enough sleep | 634 (55.7%) | 288 (25.7%) | 197 (17.6%) |
| Continuous operations | 595 (53.8%) | 299 (27.0%) | 213 (19.2%) |
| Long deployment length | 928 (84.6%) | 132 (12.0%) | 37 (3.4%) |
| Working with Afghan National Security Forces (Afghan National Army or Afghan National Police) | 736 (70.6%) | 171 (16.4%) | 136 (13.0%) |
