# Supplementary material for: Psychological distress across the deployment cycle: exploratory growth mixture model
Source: BJPsych Open. 2021 May 4;7(3):e89. doi: 10.1192/bjo.2021.50 (PMC8142545; doi:10.1192/bjo.2021.50)
Supplement: Supplementary file 1 [file bjosup.zip › S2056472421000508sup004.docx]

| Table 6 (Supplementary)  Fit Indices from unconditional modeling of depression outcome alone | | | | |
| --- | --- | --- | --- | --- |
|  | One Class | Two Classes | Three Classes^1^ | Four Classes  (Not Selected)^2^ |
| Loglikelihood | -9597.49 | -9410.89 | -9318.95 | -9268.40 |
| AIC | 19213.00 | 18845.78 | 18667.90 | 18572.80 |
| BIC | 19258.36 | 18906.27 | 18743.51 | 18663.53 |
| SSA-BIC | 19229.77 | 18868.15 | 18695.87 | 18606.36 |
| Entropy | N/A | .91 | .91 | .91 |
| LMR-LRT^3^ p-value | N/A | < .001 | .07 | .07 |
| VLMR-LRT^4^ p-value | N/A | < .001 | .07 | .07 |
| BLRT^5^ p-value | N/A | < .001 | < .001 | < .001 |
| Smallest Class | N/A | 12.1% | 6.0% | 1.1% |
| ^1^ Model selected met all *a priori* criteria  ^2^ Model rejected based on failure to meet “parsimony and interpretability” criterion  ^3^ Lo-Mendell-Rubin Likelihood Ratio Test  ^4^ Vuong-Lo-Mendell-Rubin Likelihood Ratio Test  ^5^ Bootstrap Likelihood Ratio Test | | | | |
